# Thermo‐haemodynamic coupling during regional thigh heating: Insight into the importance of local thermosensitive mechanisms in blood circulation

**DOI:** 10.1113/EP091556

**Published:** 2024-01-17

**Authors:** Nuno Koch Esteves, Jeneil McDonald, José González‐Alonso

**Affiliations:** ^1^ Division of Sport, Health, and Exercise Sciences, Department of Life Sciences, College of Health, Medicine and Lifes Sciences Brunel University London Uxbridge UK; ^2^ University College of Osteopathy London UK

**Keywords:** blood flow, haemodynamics, heat, thermal mechanisms

## Abstract

A positive relationship between local tissue temperature and perfusion exists, with isolated limb‐segment hyperthermia stimulating hyperaemia in the heated region without affecting the adjacent, non‐heated limb segment. However, whether partial‐limb segment heating evokes a heightened tissue perfusion in the heated region without directly or reflexly affecting the non‐heated tissues of the same limb segment remains unknown. This study investigated, in 11 healthy young adults, the lower limb temperature and haemodynamic responses to three levels of 1 h upper‐leg heating, none of which alter core temperature: (1) whole‐thigh (WTH; water‐perfused garment), (2) quadriceps (QH; water‐perfused garment) and (3) partial‐quadriceps (PQH; pulsed shortwave diathermy) heating. It was hypothesised that perfusion would only increase in the heated regions. WTH, QH and PQH increased local heated tissue temperature by 2.9 ± 0.6, 2.0 ± 0.7 and 2.9 ± 1.3°C (*P* < 0.0001), respectively, whilst remaining unchanged in the non‐heated hamstrings and quadriceps tissues during QH and PQH. WTH induced a two‐fold increase in common femoral artery blood flow (*P* < 0.0001) whereas QH and PQH evoked a similar ∼1.4‐fold elevation (*P* ≤ 0.0018). During QH and PQH, however, tissue oxygen saturation and laser‐Doppler skin blood flow in the adjacent non‐heated hamstrings or quadriceps tissues remained stable (*P* > 0.5000). These findings in healthy young humans demonstrate a tight thermo‐haemodynamic coupling during regional thigh heating, providing further evidence of the importance of local heat‐activated mechanisms on the control of blood circulation.

## Abstract (highlights)

- **What is the central question of the study?**
  Limb‐segment hyperthermia increases perfusion without evoking haemodynamic changes in the adjacent limb‐segment: does this hold true within a small limb‐area?
- **What is the main finding and its importance?**
  Local whole‐thigh, quadriceps and partial‐quadriceps heating increased tissue temperature, blood flow and oxygen saturation in the heated region without affecting these parameters in the non‐heated area within the same segment. These findings further support the notion that local thermosensitive mechanisms are the primary regulators of thermal hyperaemia.

## 1 INTRODUCTION

A well‐established, positive relationship exists between local tissue temperature and blood flow during whole‐body or local‐limb hyperthermia (Chiesa et al., 2016; Heinonen et al., 2011; Johnson & Proppe, 1996; Kalsi et al., 2017; Koch Esteves et al., 2021; Pearson et al., 2011). Recent research from our laboratory demonstrated that segmental‐limb heating – that is, isolated upper‐ and lower‐leg heating – produces comparable magnitudes of conduit artery hyperaemia for their respective limb segment during whole‐leg heating, without affecting tissue temperature or perfusion in the adjacent non‐heated segment (Koch Esteves et al., 2021). These findings suggest that during segmental‐limb heating, adjacent limb segments are not affected by central neural reflexes (Barcroft et al., 1947; Johnson et al., 1976) or indirect heating, whether it be through conductive or convective heat transfer via the tissues and blood, respectively (González‐Alonso, 2012; Incropera et al., 1996; Xiang & Liu, 2008).

Explanations for the observed maintenance of adjacent, non‐heated segment perfusion could be that it is due to (a) an insufficient hyperthermic stimulus to evoke reflex drives (Taylor et al., 1984) or (b) an excessively steep positive temperature gradient between the heated and non‐heated segments, whereby heat – whether via conductance in the tissues or convection in the blood – is being quickly dissipated in the normothermic limb segment as per Pennes's bioheat equation (Arkin et al., 1994; Fiala & Havenith, 2015; Incropera et al., 1996; Pennes, 1948). However, whether this phenomenon would hold true within a limb segment where the heated and non‐heated areas are in proximity remains unclear. For instance, would solely heating the whole or a portion of the quadriceps increase temperature and thus blood flow in the hamstrings and non‐heated quadriceps section or would the hyperaemia be solely confined in the heated area? Research exploring the clinical application of targeted hyperthermia on tumours has found large increases in tissue perfusion and oxygenation of the heated tissue (Bosque et al., 2021; Elming et al., 2019; Song, 1984), while the temperature of the adjacent healthy tissue remained unaltered (Kim et al., 1977; LeVeen et al., 1976; Suit & Gerweck, 1979). Although the unchanged temperature of the adjacent tissue is indicative of a maintained tissue perfusion, direct measures of macro‐ and microcirculation tissue perfusion in healthy limb tissue are required to verify or refute this assumption.

Consequently, the aim of the present study was to comprehensively assess the relationship between local limb‐segment heating and the ensuing hyperaemia, specifically investigating temperature, tissue oxygenation and haemodynamic responses in the major arteries of the human leg during whole‐thigh heating (WTH), quadriceps heating (QH) and partial‐quadriceps heating (PQH). It was hypothesised that: (a) all local hyperthermia interventions would increase the blood flow profiles of the femoral arteries which supply the upper leg, with the magnitude of hyperaemia being proportional to the volume of heated tissue, and (b) muscle and skin temperature, tissue perfusion, oxygen saturation and skin blood flow would remain unchanged in the adjacent non‐heated areas.

## 2 METHODS

### 2.1 Ethical approval

The study was approved by the Brunel University London Research Ethics Committee (38109‐MHR‐Oct/2022‐41690‐2) and was performed in accordance with the *Declaration of Helsinki*, except for registration in a database. All participants provided informed written consent prior to their participation in the present study following a detailed written and verbal explanation of the experimental protocol.

### 2.2 Participants

Eleven healthy, physically active adults (4 women) participated in the present study. Participants had a mean ± SD age of 22 ± 6 years, a height of 174.4 ± 8.8 cm and body mass of 76.1 ± 13.1 kg (Table 1). Participants were considered healthy and physically active following the completion of a health questionnaire and a basic cardiovascular screening. Participants refrained from heavy exercise for 48 h, alcohol consumption for 24 h and caffeine consumption for 12 h before the commencement of the protocols. Moreover, female participants were requested to avoid scheduling their laboratory visit during their menses.

**TABLE 1:** Participant demographic and anthropometric characteristics.

Variable	Value
Age (years)	22 ± 6
Sex (*n* (%))	
Female	4 (36)
Male	7 (64)
Height (cm)	174.4 ± 8.8
Mass (kg)	76.1 ± 13.1
Right leg volume (l)	12.2 ± 2.4
Right leg lean volume (%)	74.7 ± 9.1
Right leg non‐lean volume (%)	25.3 ± 9.1
Left leg volume (l)	12.2 ± 2.3
Left leg lean volume (%)	74.9 ± 9.1
Left leg non‐lean volume (%)	25.1 ± 9.1

Values are means ± SD, except where stated otherwise, for 11 participants.

### 2.3 Experimental protocols

**FIGURE 1:**
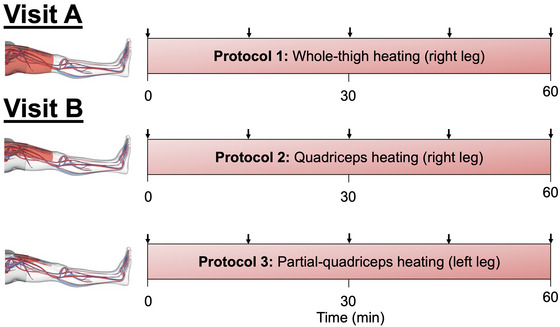
Schematic representation of experimental protocol. Arrows illustrate the times in which an ultrasound blood flow measurement was conducted. Blood flow was measured at the common, superficial and profunda femoral arteries and popliteal artery. Core temperature, leg muscle and skin temperatures, leg tissue oxygen saturation, leg skin blood flow and central haemodynamics were measured continuously throughout the protocol. Protocol 1 was conducted during visit A, and protocols 2 and 3 on visit B. The respective visits were randomised and counterbalanced.

The present study consisted of three protocols: (a) WTH, (b) QH and (c) PQH, which were conducted over two visits (Figure 1). Protocol 1 was completed during visit A whilst protocols 2 and 3 were completed during another visit, B, with the order being counterbalanced among participants. Upon arriving at the laboratory, participants were asked to weigh themselves in a semi‐nude state and then had their height measured (Seca 798 scale, Hamburg, Germany) and in the case for visit 1 only, had their leg anthropometric measurements recorded. The latter data allowed an estimate of leg composition using the method reported by Jones and Pearson (1969). Seven leg circumferences were taken at the gluteal furrow, one‐third subischial (one‐third of the distance between the gluteal furrow and the popliteal crease), the minimum circumference above the knee, the maximum circumference at the knee joint, the minimum circumference below the knee, the maximum circumference at the calf and the minimum circumference at the ankle joint. Additionally, skinfold measurements were obtained at the following four sites: one‐third subischial (anterior and posterior sites) and at the maximum calf circumference (lateral and medial sites) using skinfold callipers (Jones & Pearson, 1969). Subsequently, participants sat on a custom‐built bed within a climate chamber room set at an ambient temperature and humidity of 22°C and 30−40%, respectively.

#### 2.3.1 Protocol 1: effects of WTH

Participants were instrumented with ECG electrodes, temperature thermistors, tissue oxygenation optode pads and the finometer upper‐arm and middle finger cuffs (described below). The experimental protocol was initiated with baseline haemodynamic measurements of the common femoral artery (CFA), superficial femoral artery (SFA), profunda (deep) femoral artery (PFA) and popliteal artery (POA) in the right leg. Next, participants were fitted with a custom‐made water‐perfusion trouser on their right upper leg, which was then wrapped in a survival blanket to optimise the heating procedure by limiting heat loss from the trouser to the surrounding environment. The trouser was connected to a thermostatically controlled water circulator (Julabo F‐34, Seelbach, Germany), which continuously circulated water at a temperature of 50°C. During the 1 h heating protocol, blood flow was measured every 15 min at the CFA, SFA, PFA and POA of the right, experimental leg (Figure 1).

#### 2.3.2 Protocol 2: effects of QH

Following instrumentation of ECG electrodes, temperature thermistors, tissue oxygenation optode pads and finometer cuffs – much like protocol 1 – participants had baseline haemodynamic measurements of the CFA, SFA, PFA and POA in the right leg. Subsequently, participants were fitted with a custom‐made water‐perfusion trouser which solely covered the top of their right upper leg (i.e. the quadriceps). A survival blanket was then placed on top of the heated trouser to limit heat loss; however, care was taken not to cover the remainder of the upper leg (i.e. the hamstrings) which was left exposed. The trouser was heated as described in protocol 1. During the 1 h heating protocol, blood flow was measured every 15 min at the CFA, SFA, PFA and POA of the right, experimental leg (Figure 1).

#### 2.3.3 Protocol 3: effects of PQH

This protocol followed immediately after the completion of protocol 2. Baseline haemodynamic measurements of the CFA, SFA, PFA and POA were recorded in the left leg. PQH was conducted using a pulsed shortwave diathermy (MegaPulse II, EMS Physio, Wantage, UK) at 800 pulses per second, with a pulse duration of 400 μs. The heating drum has a surface area of ∼200 cm^2^. Thus, if one assumes that whole‐thigh and quadriceps hyperthermia heat 100 and 50% of total upper‐leg surface area, respectively, using the anthropometric measures calculated from the present cohort, partial‐quadriceps hyperthermia heated ∼8% of the upper‐leg (i.e. 16% of the quadriceps). During the 1‐h heating protocol, blood flow was measured every 15 min at the CFA, SFA, PFA and POA of the left, experimental leg (Figure 1).

### 2.4 Temperature measurements

Core temperature (*T*_c_) was measured using a rectal probe (Ret‐1 Special, Physitemp, Clifton, NJ, USA) which was self‐inserted 15 cm past the sphincter muscle. Tympanic temperature (*T*_Tym_) was measured using a commercially available thermometer (Thermoscan 7, Braun, Germany). Skin temperature (*T*_sk_) was measured in the quadriceps at two different locations (one proximal and one distal) and the hamstrings of the experimental leg using commercially available thermistors (IT‐18, Physitemp, Clifton, NJ, USA) which were securely held in place using medical tape. Muscle temperature (*T*_m_) in the proximal portion of the vastus lateralis muscle (during all three protocols), the distal portion of the vastus lateralis muscle (during protocol 3), and in the middle of the biceps femoris muscle of the right leg (during protocols 1 and 2) was measured using thermistors (T‐204f, Physitemp) inserted via an 18G catheter ∼3 cm deep into the muscle. *T*_c_, *T*_sk_ and *T*_m_ were recorded online using a commercially available thermocouple metre (TC‐2000, Sable Systems International, Las Vegas, NV, USA) collected at 1000 Hz through a data acquisition system (PowerLab 26T, ADInstruments, Dunedin, New Zealand), and exported in 30‐s bins using a commercially available data acquisition software (LabChart 7, ADInstruments). Data were then imported and analysed in Microsoft Excel software, and reported as 2‐min averages. Temperature data are reported every 5 min for the first 15 min of protocols 1 and 2 to better characterise the sharp increase in tissue temperatures and then every 15 min, in parallel with haemodynamic measurements, for the remainder of the protocols. During protocol 3, temperature data are reported at baseline, 30 and 60 min due to interference between the thermocouple metre and diathermy unit. In addition, mean tissue temperature for the heated regions was calculated using:

$$\begin{array}{ccc} \;{\bar{T}}_{\mathrm{Whole}-\mathrm{thigh}} & = & \left(\frac{T_{m,\mathrm{quad}1}+T_{m,\mathrm{ham}}\;}{2}\times 0.92\right)\;\\ & & +\left(\frac{T_{\mathrm{sk},\mathrm{quad}1}+T_{\mathrm{sk},\mathrm{quad}2}+T_{\mathrm{sk},\mathrm{ham}}\;}{3}\times 0.08\right),\\ & & {\bar{T}}_{\mathrm{Quadriceps}}=\left(T_{m,\mathrm{quad}1}\times 0.92\right)\;\\ & & +\left(\frac{T_{\mathrm{sk},\mathrm{quad}1}+T_{\mathrm{sk},\mathrm{quad}2}\;}{2}\times 0.08\right)\;\text{and}\;{\bar{T}}_{\mathrm{Partial}-\mathrm{quadriceps}}\\ & & =\left(T_{m,\mathrm{quad}1}\times 0.92\right)\;+\left(T_{\mathrm{sk},\mathrm{quad}1}\times 0.08\right), \end{array}$$

where quad1, quad2 and ham represent the proximal and distal portions of the quadriceps and hamstrings, respectively. Mean tissue temperature formulas were created using previously reported volume ratios of the different tissue compartments in the leg (Wang et al., 1999).

### 2.5 Haemodynamic measurements

Heart rate was continuously measured using a three‐lead echocardiogram. Also, arterial blood pressure was measured non‐invasively – at the same time points as arterial blood flow measurements – using infrared photoplethysmography (Finometer, Finapres Medical Systems, Enschede, Netherlands), through a cuff on the middle finger of the right hand. Blood flow was measured at set time points (Figure 1) – recording two 12‐s Doppler images – throughout the protocols in the various arteries using a duplex Doppler ultrasound system (Vivid E95, GE Medical Systems, Little Chalfont, UK) with a 9‐MHz linear array transducer probe (GE Medical Systems) at an insonation angle of ≤60°, with sample volume positioned in the centre of the artery. Before commencing baseline blood flow measures, arterial sites for the CFA, SFA, PFA and POA, in the right leg during protocol 1 and both legs for protocols 2 and 3, were located and marked to ensure blood flow measures which were consistently measured at the same site. SFA and PFA blood flow measurements were acquired at a distance of ≥2 cm from the femoral bifurcation to prevent turbulent flow disruption to the measurements, and thus improve validity of measures. Blood flow (ml min^−1^) was calculated using the following equation: $\mathrm{BF}\;=V_{\mathrm{mean}}\;\times \pi \;\times {\left(\frac{D_{\mathrm{mean}}}{2}\right)}^{2}\;\times \;60$, where *V*_mean_ is the average centreline blood velocity (cm s^−1^) and *D*_mean_ (cm) is the average internal diameter calculated using: $D_{\mathrm{mean}}=\frac{1}{3}\;\left(D_{\mathrm{systole}}\right)+\;\frac{2}{3}\left(D_{\mathrm{diastole}}\right)$ (Rådegran, 1997). Furthermore, upper‐leg blood flow was calculated as the difference between whole‐leg blood flow (CFA) and lower‐leg blood flow (POA).

Shear rate (SR) was calculated using: $\mathrm{SR}\;=\frac{4\;\times \;V_{\mathrm{mean}}}{D_{\mathrm{mean}}}\;,$ where *V*_mean_ is mean blood velocity. Additionally, vascular conductance (VC) was calculated using: VC=BF/MAP, represented as ml min^−1^ mmHg^−1^, BF is blood flow (ml min^−1^) and MAP is mean arterial pressure (mmHg). Blood flow was analysed offline using a commercially available software (EchoPAC, GE Medical, Horton, Norway). Blood velocity was averaged over two 12‐s Doppler images, and average diameter was determined from four 2D B‐mode images. Furthermore, central haemodynamic data were collected at 1000 Hz using a commercially available data acquisition system (PowerLab 26T, ADInstruments) and exported in 30‐s bins using a commercially available data acquisition software (LabChart 7, ADInstruments). Following exportation, data were imported and analysed in Microsoft Excel software. Data are reported as 2‐minute averages throughout the three protocols. Furthermore, quadriceps skin blood flow was measured in all three protocols via laser‐Doppler flowmetry (PeriFlux Flowmetry System, Perimed, Järfälla, Denmark), reported in perfusion units (PU). The probe was attached to the skin of the thigh, specifically on the distal portion of the vastus lateralis. During protocols 1 and 2, skin blood flow was measured under the heated region; however, during protocol 3, it was measured in the non‐heated area of the quadriceps.

### 2.6 Tissue oxygen saturation measures

Direct and continuous measurements of regional tissue haemoglobin oxygen saturation were obtained in the experimental upper legs using a near‐infrared spectroscopy unit (NIRS; INVOS 5100C Cerebral Oximeter; Somanetics Corp, Troy, MI, USA). The optodes were placed on the skin surrounding the quadriceps and hamstrings of the experimental upper leg, with the same positioning as the *T*_sk_ thermistors, and taped to reduce interference from external light sources. It was not possible to measure tissue oxygen saturation at the heated region during protocol 3 – that is, proximal quadriceps under the diathermy unit – due to interference between the near‐infrared spectroscopy and diathermy units.

### 2.7 Statistical analysis

Statistical analysis was conducted using R Studio (version 2022.07.1+554, Team (2022)). An independent Student's *t*‐test was conducted to identify any differences in anthropometric data between the right and left leg. In addition, linear mixed‐effects models were employed to investigate differences between protocols and over time in all measured variables – that is, central and local haemodynamics, temperature and tissue oxygenation – during the three heating protocols. The linear mixed‐effects models were conducted following the confirmation of the data's normality via the Shapiro–Wilk test and Mauchly's test of sphericity. Following the linear mixed‐effects models, once a significant interaction between protocol and time was found, a Bonferroni *post hoc* test was conducted to locate the specific time points at which those changes occurred. Significance was set at *P* < 0.05. Note, if no time points or protocols are specified, *P‐*values were with reference to the interaction (time × protocol) following the linear mixed‐effects model; if time points or protocols are specified, then *P‐*values were obtained from the *post hoc* test. Results are expressed as means ± SD, with all data corresponding to the experimental legs. Moreover, linear, exponential and polynomial regression curve fit tests were performed using GraphPad Prism (version 8, GraphPad Software, La Jolla, CA, USA) to assess the relationships among various key data, with *R* ^2^ representing the goodness of fit. Subsequently, Akaike's information criterion was used to evaluate which model provides the most appropriate fit.

## 3 RESULTS

### 3.1 Demographic and anthropometric characteristics

Demographic and anthropometric data for the participants are reported in Table 1. No differences were observed in leg volume (*P* = 0.748) or proportion of lean to non‐lean mass (*P* = 0.797) between the right and left legs.

### 3.2 Regional and core temperature responses to whole‐thigh, quadriceps and partial‐quadriceps heating

**FIGURE 2:**
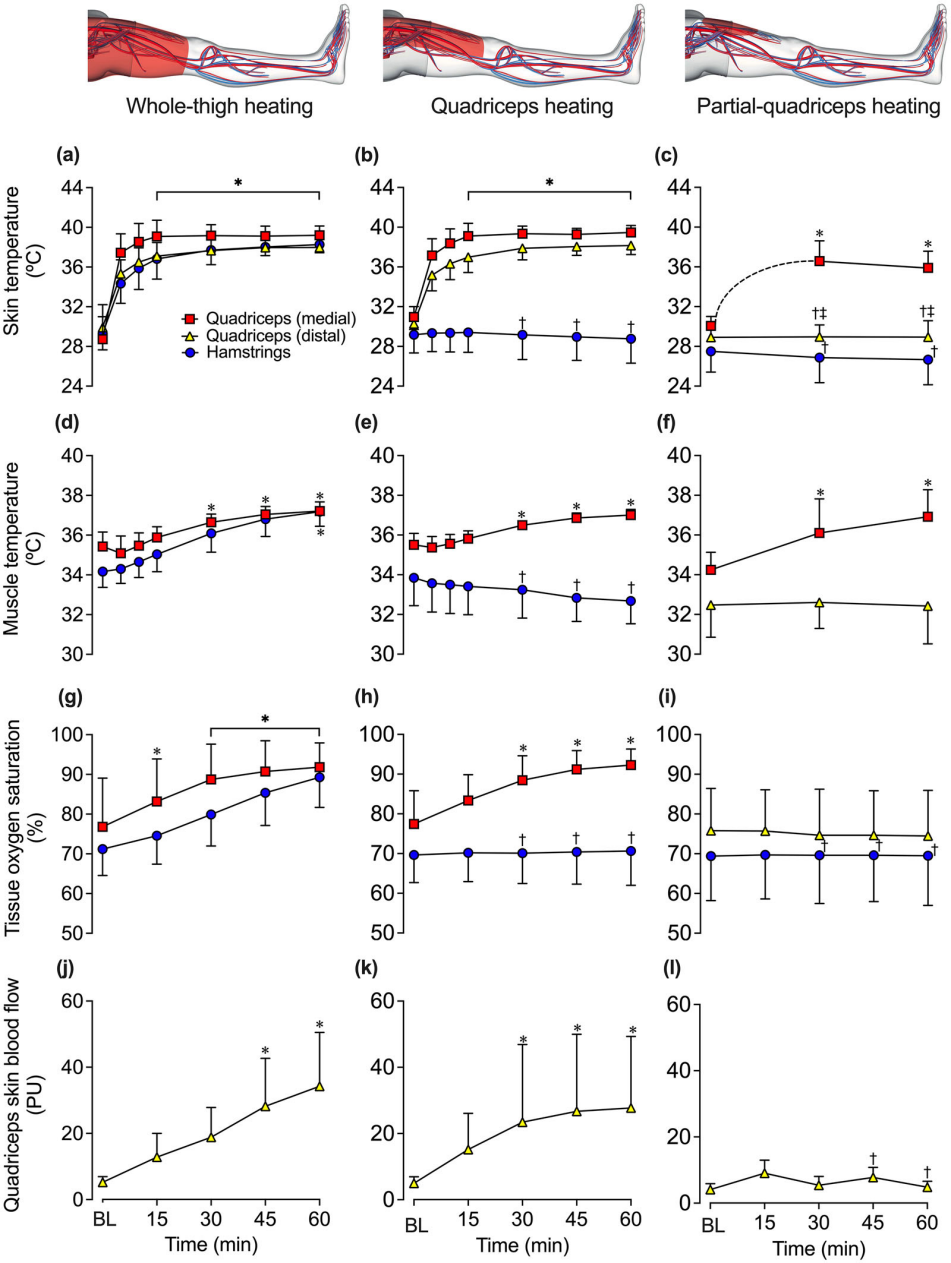
Skin and muscle leg temperatures (a–f), regional tissue oxygen saturation (g–i) and skin blood flow (j–l) during whole‐thigh (a, d, g, j), quadriceps (b, e, h, k) and partial‐quadriceps (c, f, i, l) hyperthermia. Data represented as means ± SD (*n* = 11). BL signifies baseline measurements. Due to the fewer data points recorded during partial‐quadriceps heating, a curved dotted line between BL and 30 min quadriceps (medial) skin temperature data points was plotted to illustrate the predicted change in temperature over time based on the literature (Hafen et al., 2018). Quadriceps regional tissue oxygenation and skin blood flow were measured within the heated area during protocols 1 and 2; however, it was only measured outside the heated proximal quadriceps area during protocol 3. *Different from baseline across time within the same protocol, *P* < 0.05. †Different from whole‐thigh heating, *P* < 0.05. ‡Different from quadriceps heating, *P* < 0.05.

Leg muscle and skin temperatures are illustrated in Figure 2, whilst core and tympanic temperatures are reported in Table 2. As per experimental design, all three heating protocols induced significant increases in tissue temperature of their respective heated regions (*P* = 0.0145), whilst the measured unheated regions during QH and PQH remained unchanged (Figure 2). Specifically, WTH increased ${\bar{T}}_{\mathrm{Whole}-\mathrm{thigh}}$ to 37.3 ± 0.4°C (∆ = +2.9 ± 0.6°C; *P* < 0.0001), QH increased ${\bar{T}}_{\mathrm{Quadriceps}}$ to 37.2 ± 0.3°C (∆ = +2.0 ± 0.7°C; *P* < 0.0001) and PQH increased ${\bar{T}}_{\mathrm{Partial}-\mathrm{quadriceps}}$ to 36.8 ± 1.3°C (∆ = +2.9 ± 1.3°C; *P* < 0.0001), with these temperatures representing the average tissue temperature of the respective heated regions. No between‐protocol differences were observed in the tissue temperature of the heated regions. Moreover, core temperature was stable during QH and PQH but decreased marginally following 1 h of WTH protocol (∆ = −0.2 ± 0.3; *P* < 0.001) (Table 2). No between‐protocol differences in core temperature were observed (Table 2). Lastly, no differences across time or between protocols were observed in tympanic temperature (*P* = 0.2664) (Table 2).

**TABLE 2:** Influence of whole‐thigh, quadriceps and partial‐quadriceps heating on body temperature, and central haemodynamics.

	Time (min)				
Variable	0	15	30	45	60
*T*_c_ (°C)					
Whole‐thigh heating	37.2 ± 0.2	37.0 ± 0.2	37.0 ± 0.2*	37.0 ± 0.3*	37.0 ± 0.3*
Quadriceps heating	37.1 ± 0.2	36.9 ± 0.2	36.9 ± 0.2	36.9 ± 0.2	36.9 ± 0.2
Partial‐quadriceps heating	36.9 ± 0.2	—	36.9 ± 0.2	—	37.1 ± 0.2
*T*_Tym_ (°C)					
Whole‐thigh heating	36.9 ± 0.2	36.8 ± 0.2	36.8 ± 0.2	36.7 ± 0.2	36.7 ± 0.2
Quadriceps heating	36.7 ± 0.4	36.7 ± 0.3	36.7 ± 0.4	36.7 ± 0.4	36.6 ± 0.4
Partial‐quadriceps heating	36.7 ± 0.4	—	36.7 ± 0.4	—	36.7 ± 0.4
HR (beats min^−1^)					
Whole‐thigh heating	64 ± 7	65 ± 8	64 ± 8	65 ± 7	66 ± 6
Quadriceps heating	60 ± 8	59 ± 7	60 ± 7	59 ± 8	59 ± 8
Partial‐quadriceps heating	59 ± 9	57 ± 6	59 ± 8	58 ± 8	62 ± 8
MAP (mmHg)					
Whole‐thigh heating	87 ± 8	89 ± 8	89 ± 10	88 ± 14	90 ± 13
Quadriceps heating	92 ± 13	88 ± 13	90 ± 11	92 ± 11	90 ± 10
Partial‐quadriceps heating	93 ± 10	93 ± 11	97 ± 11	98 ± 14	95 ± 13

Values are means ± SD for 11 participants. *Different from baseline, *P* < 0.05. Abbreviations: HR, heart rate; MAP, mean arterial pressure; *T*_c_, core temperature; *T*_Tym_, tympanic temperature.

### 3.3 Leg haemodynamics, tissue oxygen saturation and systemic haemodynamics during whole‐thigh, quadriceps and partial‐quadriceps heating

**FIGURE 3:**
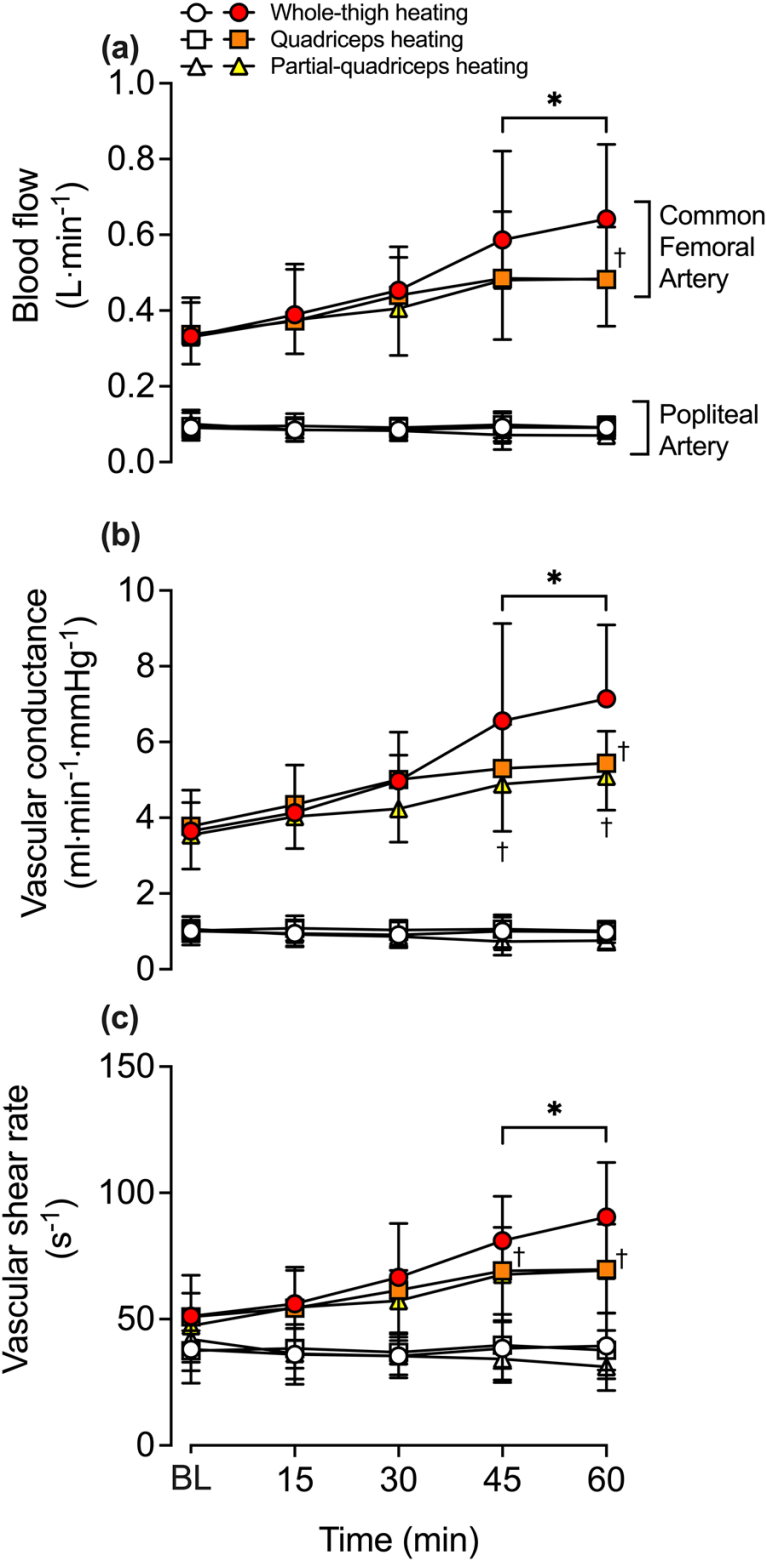
Blood flow (a), vascular conductance (b) and shear rate (c) during whole‐thigh, quadriceps and partial‐quadriceps hyperthermia. Data represented as means ± SD (*n* = 11) for the common femoral (CFA) and popliteal (POA) arteries, represented by filled and open symbols, respectively. BL signifies baseline measurements. *Different from baseline across time within the same protocol, *P* < 0.05. †Different from whole‐thigh heating.

**FIGURE 4:**
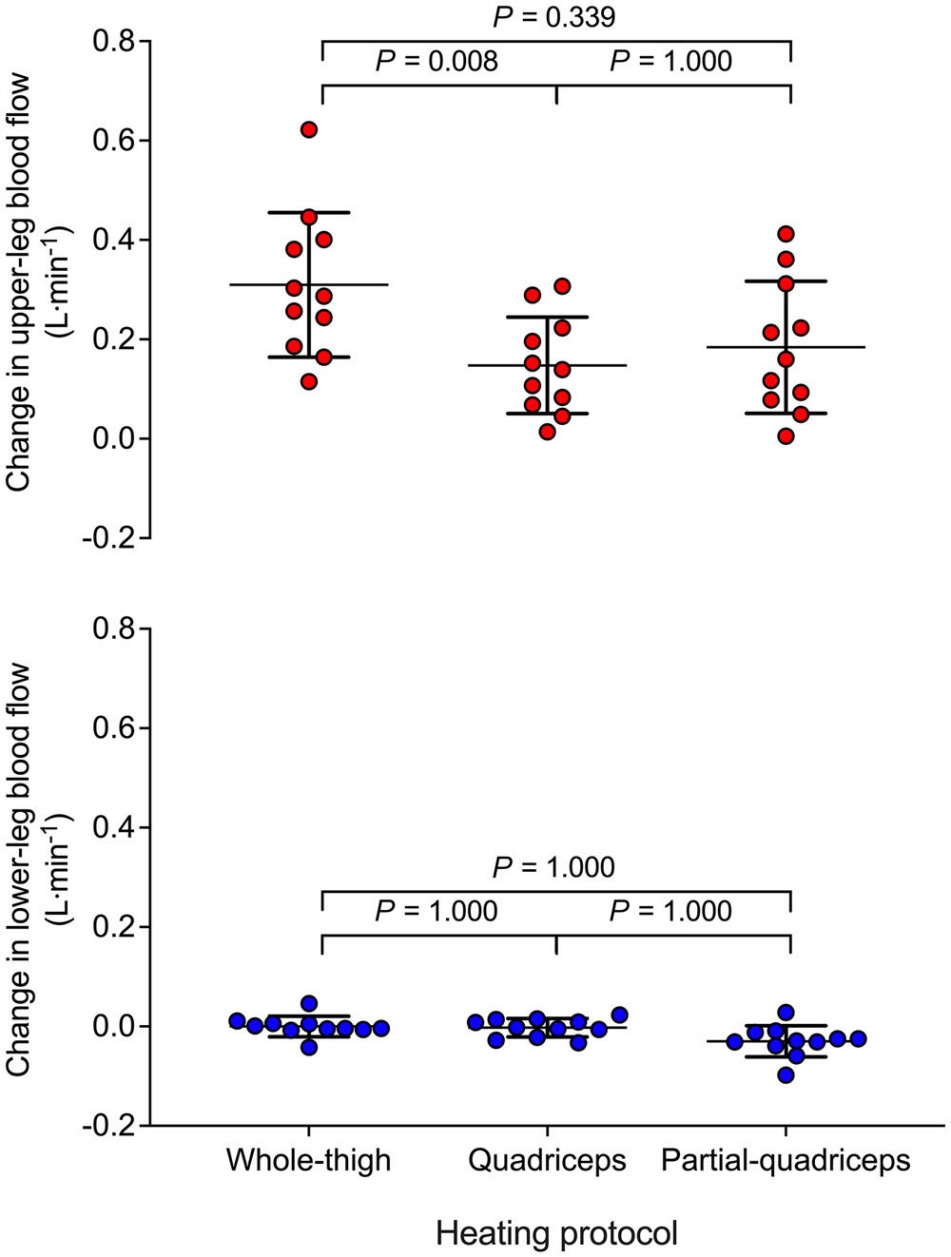
Individual changes in regional blood flow following 1 h whole‐thigh, quadriceps and partial‐quadriceps hyperthermia. Circles depict the individual data points while the lines illustrate means ± SD (*n* = 11). Red circles represent heated segments while blue circles represent control segment.

**FIGURE 5:**
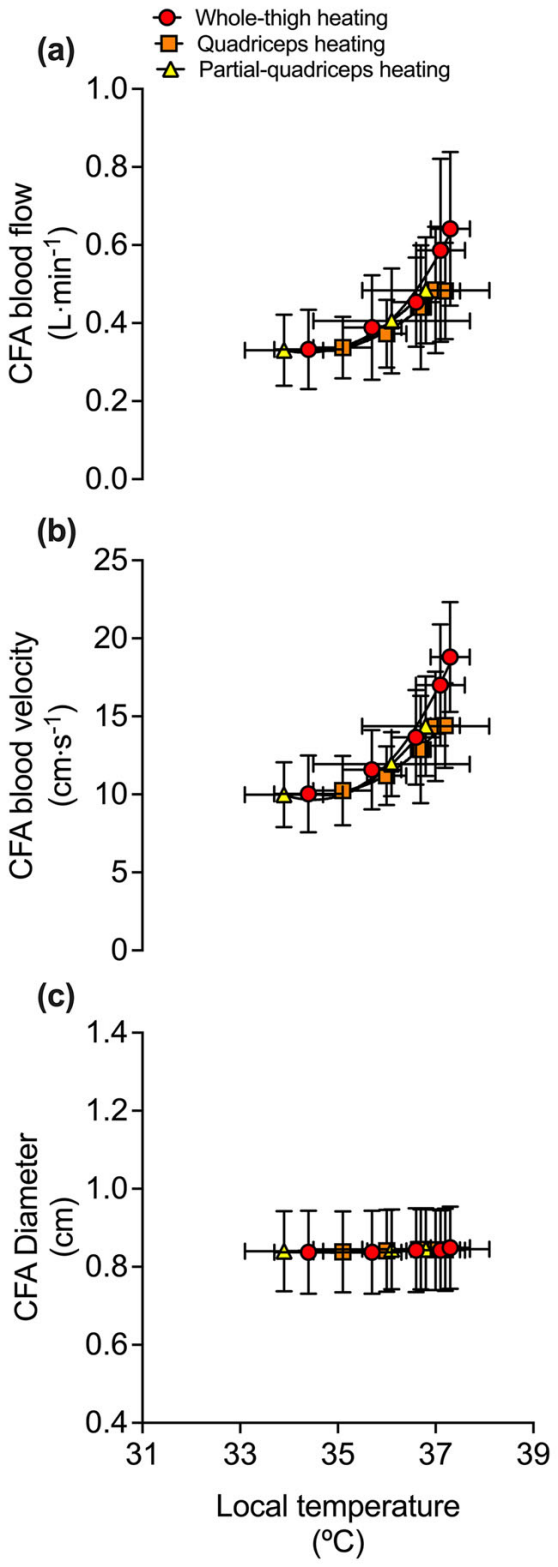
Relationship between the mean local tissue temperature and common femoral artery (CFA) blood flow (a), blood velocity (b) and arterial diameter (c) during whole‐thigh, quadriceps and partial‐quadriceps hyperthermia. Local temperature signifies the corresponding average heated tissue temperature for the protocol, as discussed in the methods – that is, ${\bar{T}}_{\mathrm{Whole}-\mathrm{thigh}}$, ${\bar{T}}_{\mathrm{Quadriceps}}$ and ${\bar{T}}_{\mathrm{Partial}-\mathrm{quadriceps}}$. Data represented as means ± SD (*n* = 11). Vertical error bars signify blood flow SD, while horizontal error bars signify local temperature SD. Lines represent the exponential fit of the data.

Complete haemodynamic responses during 1 h of WTH, QH and PQH for the CFA, SFA, PFA and POA are reported in Table 3 and Figure 3. Femoral artery blood flow increased steadily during WTH with CFA, SFA and PFA blood flow increasing ∼2‐fold above baseline (∆ = +0.31 ± 0.16, +0.13 ± 0.12 and +0.13 ± 0.07 l min^−1^, respectively) following 1 h (all *P* < 0.0001). One hour of QH resulted in a smaller magnitude of upper‐leg tissue perfusion in comparison to WTH (*P* = 0.0080; Figure 4) with CFA blood flow increasing ∼1.4‐fold (∆ = +0.15 ± 0.09 l min^−1^, *P* = 0.0018); however, no significant changes were observed in SFA (*P* = 0.0836) and PFA (*P* = 0.5546) blood flow. PQH increased CFA and PFA blood flow by ∼1.4−1.6‐fold (∆ = +0.15 ± 0.12 and +0.07 ± 0.06 l min^−1^, *P* = 0.0006 and *P* = 0.0002, respectively) whilst SFA blood flow remained unchanged (*P* = 1.000). These increases in upper‐leg blood flow were not lower than those observed during WTH (*P* = 0.3392) and QH (*P* = 1.0000) (Figure 4). During all thigh heating protocols, POA blood flow remained unchanged (*P* = 0.0642) (Figure 4). Whole‐leg blood flow was related (second‐order polynomial) to increases in mean tissue temperature of the heated region during WTH (*R* ^2^ = 0.35), QH (*R* ^2^ = 0.19) and PQH (*R* ^2^ = 0.22) (Figure 5). No relationship was observed between mean tissue temperature and diameter (Figure 5); as such, no changes in diameter were observed in the CFA (*P* = 0.4231), SFA (*P* = 0.6718) and PFA (*P* = 0.0642). Correspondingly, relationships (second order polynomial) were observed between blood velocity and mean tissue temperature during WTH (*R* ^2^ = 0.54), QH (*R* ^2^ = 0.27) and PQH (*R* ^2^ = 0.36) (Figure 5), which mirrored the changes in blood flow. Moreover, quadriceps skin blood flow increased ∼6.6‐fold (*P* < 0.0001) and ∼5.6‐fold (*P* < 0.0001) during WTH and QH, respectively, but remained unchanged during PQH (*P* = 1.0000) as the optode was placed outside the heated area (Figure 2).

**TABLE 3:** Influence of whole‐thigh, quadriceps and partial‐quadriceps heating on leg haemodynamics.

	Time (min)				
Variable	0	15	30	45	60
CFA blood flow (l min^−1^)					
Whole‐thigh heating	0.33 ± 0.10	0.39 ± 0.13	0.45 ± 0.11*	0.59 ± 0.23*	0.64 ± 0.20*
Quadriceps heating	0.34 ± 0.08	0.37 ± 0.09	0.44 ± 0.16	0.48 ± 0.16*	0.48 ± 0.12*^†^
Partial‐quadriceps heating	0.33 ± 0.09	0.38 ± 0.13	0.41 ± 0.13	0.48 ± 0.18*	0.48 ± 0.14*
SFA blood flow (l min^−1^)					
Whole‐thigh heating	0.17 ± 0.03	0.19 ± 0.06	0.22 ± 0.05	0.26 ± 0.11*	0.30 ± 0.12*
Quadriceps heating	0.17 ± 0.04	0.20 ± 0.06	0.19 ± 0.06	0.23 ± 0.10	0.23 ± 0.07^†^
Partial‐quadriceps heating	0.18 ± 0.06	0.19 ± 0.05	0.19 ± 0.06	0.19 ± 0.6	0.21 ± 0.08^†^
PFA blood flow (l min^−1^)					
Whole‐thigh heating	0.12 ± 0.04	0.15 ± 0.07	0.17 ± 0.07*	0.23 ± 0.09*	0.25 ± 0.10*
Quadriceps heating	0.12 ± 0.04	0.12 ± 0.04	0.14 ± 0.04	0.14 ± 0.03^†^	0.16 ± 0.04^†^
Partial‐quadriceps heating	0.11 ± 0.03	0.13 ± 0.07	0.14 ± 0.05	0.16 ± 0.05	0.18 ± 0.05*
POA blood flow (l min^−1^)					
Whole‐thigh heating	0.09 ± 0.02	0.09 ± 0.03	0.08 ± 0.03	0.09 ± 0.04	0.09 ± 0.03
Quadriceps heating	0.09 ± 0.04	0.10 ± 0.03	0.09 ± 0.03	0.10 ± 0.03	0.09 ± 0.03
Partial‐quadriceps heating	0.10 ± 0.04	0.09 ± 0.03	0.08 ± 0.02	0.08 ± 0.03	0.07 ± 0.02
CFA vascular conductance (ml min^−1^ mmHg^−1^)					
Whole‐thigh heating	3.6 ± 1.1	4.1 ± 1.3	5.0 ± 1.3	6.6 ± 2.6*	7.1 ± 1.9*
Quadriceps heating	3.8 ± 1.1	4.3 ± 1.2	5.0 ± 1.6	5.3 ± 1.7*	5.4 ± 1.2*^†^
Partial‐quadriceps heating	3.5 ± 0.9	4.0 ± 1.4	4.2 ± 1.4	4.9 ± 1.6^†^	5.1 ± 1.2*^†^
SFA vascular conductance (ml min^−1^ mmHg^−1^)					
Whole‐thigh heating	1.9 ± 0.4	2.0 ± 0.6	2.5 ± 0.6	3.0 ± 1.3*	3.3 ± 1.2*
Quadriceps heating	1.9 ± 0.5	2.3 ± 0.7	2.2 ± 0.7	2.5 ± 1.0	2.6 ± 0.6^†^
Partial‐quadriceps heating	2.0 ± 0.6	2.0 ± 0.5	2.0 ± 0.5	2.0 ± 0.5^†^	2.2 ± 0.8^†^
PFA vascular conductance (ml min^−1^ mmHg^−1^)					
Whole‐thigh heating	1.3 ± 0.5	1.5 ± 0.7	1.9 ± 0.8*	2.5 ± 1.2*	2.7 ± 1.0*
Quadriceps heating	1.3 ± 0.5	1.5 ± 0.5	1.6 ± 0.5	1.6 ± 0.5^†^	1.8 ± 0.5^†^
Partial‐quadriceps heating	1.2 ± 0.3	1.4 ± 0.6	1.4 ± 0.5	1.6 ± 0.5^†^	1.9 ± 0.4*
POA vascular conductance (ml min^−1^ mmHg^−1^)					
Whole‐thigh heating	1.0 ± 0.3	0.9 ± 0.3	0.9 ± 0.3	1.0 ± 0.4	1.0 ± 0.3
Quadriceps heating	1.0 ± 0.4	1.1 ± 0.3	1.0 ± 0.3	1.1 ± 0.3	1.0 ± 0.2
Partial‐quadriceps heating	1.1 ± 0.3	0.9 ± 0.3	0.9 ± 0.3	0.7 ± 0.4	0.8 ± 0.3
CFA shear rate (s^−1^)					
Whole‐thigh heating	51 ± 16	56 ± 15	67 ± 21	81 ± 18*	90 ± 22*
Quadriceps heating	51 ± 15	54 ± 13	62 ± 17	69 ± 20*	70 ± 17*^†^
Partial‐quadriceps heating	48 ± 13	55 ± 15	57 ± 12	68 ± 19*	69 ± 18*
SFA shear rate (s^−1^)					
Whole‐thigh heating	53 ± 19	56 ± 16	68 ± 20	77 ± 24*	87 ± 30*
Quadriceps heating	56 ± 17	59 ± 13	57 ± 15	69 ± 20	68 ± 16
Partial‐quadriceps heating	55 ± 14	56 ± 13	57 ± 11	58 ± 14	62 ± 20^†^
PFA shear rate (s^−1^)					
Whole‐thigh heating	63 ± 16	73 ± 22	86 ± 22	113 ± 29*	126 ± 39*
Quadriceps heating	62 ± 17	66 ± 14	74 ± 16	77 ± 19	87 ± 29^†^
Partial‐quadriceps heating	60 ± 14	68 ± 16	74 ± 27	88 ± 29	102 ± 41*
POA shear rate (s^−1^)					
Whole‐thigh heating	38 ± 13	36 ± 12	35 ± 9	39 ± 13	39 ± 13
Quadriceps heating	37 ± 8	38 ± 8	37 ± 5	40 ± 9	38 ± 8
Partial‐quadriceps heating	42 ± 9	36 ± 10	35 ± 7	34 ± 8	31 ± 9

Values are means ± SDs for 11 participants. *Different from baseline, *P* < 0.05. ^†^Different from whole‐thigh heating, *P* < 0.05. Abbreviations: CFA, common femoral artery; PFA, profunda femoral artery; POA, popliteal artery; SFA, superficial femoral artery.

Similar responses to blood flow were observed in VC and SR during the three heating protocols (Figure 5 and Table 3). Following 1 h of WTH, VC and SR increased ∼1.7−2.1‐fold and ∼1.6−2‐fold, respectively, in all three femoral arteries (all *P* < 0.0001). During QH, CFA VC and SR increased ∼1.4‐fold (*P* = 0.0019 and *P* = 0.0077), respectively, and was lower than WTH (*P* = 0.0050 and 0.0130). However, during QH, no differences in SR across time were observed in the SFA and PFA (*P* ≥ 0.1401) and as such, SFA and PFA SR and PFA VC were lower than WTH (all *P* < 0.05) whilst SFA VC was not (*P* = 0.1328). Conversely, PQH instigated ∼1.4 increases in CFA VC (*P* = 0.0033) and SR (*P* = 0.0006), and ∼1.6 in PFA VC (*P* = 0.0005) and SR (*P* < 0.0001). VC and SR were lower during PQH in comparison to whole‐thigh for all arteries (*P* < 0.05) apart from CFA and PFA SR which were not different (*P* = 0.2807 and *P* = 0.4048, respectively). No differences, however, were observed between QH and PQH (all *P* = 1.0000).

Tissue oxygen saturation responses are illustrated in Figure 3. During WTH, quadriceps and hamstrings tissue oxygen saturation increased steadily, peaking at 92 ± 6% units (∆ = +15 ± 8% units, *P* < 0.0001) and 89 ± 8% units (∆ = +18 ± 5% units, *P* < 0.0001), respectively. Similarly, following 1 h of QH, quadriceps tissue oxygen saturation increased to 92 ± 4% units (∆ = +15 ± 6% units, *P* < 0.0001), which was similar to that observed during WTH (*P* = 1.000). Conversely, hamstring tissue oxygen saturation remained unchanged during QH (*P* = 1.000), which differed from the responses observed during WTH (*P* < 0.0001). There were no changes observed in quadriceps (*P* = 0.5270) and hamstring (*P* = 0.9960) tissue oxygen saturation during PQH, as both sites were measured outside the heated area. As such, quadriceps tissue oxygen saturation was lower following 1 h of PQH in comparison to WTH (*P* < 0.0001) and QH (*P* < 0.0001). Similarly, hamstring tissue oxygen saturation was lower following 1 h of PQH in comparison to WTH (*P* < 0.0001) but not different from QH (*P* = 1.000). Lastly, at the systemic haemodynamic level, no differences in heart rate and mean arterial pressure were observed during or between any of the three heating protocols (*P* = 0.1689 and *P* = 0.7958, respectively) (Table 2).

## 4 DISCUSSION

The present study explored the relationship between local tissue temperature and perfusion during three levels of regional thigh heating to gain further insight into the importance of local thermosensitive mechanisms in the control of blood circulation. As per the study's primary hypothesis, all heating protocols increased CFA blood flow, with WTH evoking a larger magnitude of hyperaemia than QH, but surprisingly, not greater than PQH. Moreover, in line with the secondary hypothesis, tissue temperature, blood flow and oxygen saturation remained unchanged in the respective non‐heated hamstrings, quadriceps and lower‐leg segment. Together, the present findings demonstrate a close coupling among tissue perfusion, oxygen saturation and temperature during regional thigh heating, which further substantiates the local origin of the mechanisms involved in the control of blood circulation during isolated hyperthermia.

### 4.1 Influence of regional thigh heating on tissue thermo‐haemodynamics

In this study, all three modalities of upper‐leg heating – WTH, QH and PQH – evoked sustained 1.4−2‐fold increases in CFA blood flow (Figure 3). In line with previous studies, local hyperthermia‐induced increases in leg tissue perfusion, assessed via CFA blood flow, occurred in relation to increases in local tissue temperature (Chiesa et al., 2016; Koch Esteves et al., 2021, 2023). A previous study from our laboratory revealed that the magnitude of hyperaemia was associated with the volume of the heated limb segment, with whole‐leg heating evoking the largest degree of hyperaemia, proportionally followed by upper‐ and lower‐leg heating, respectively (Koch Esteves et al., 2021). The present study extends those findings by showing that the hyperaemia during 1 h QH and WTH (+0.15 and +0.31 l min^−1^, respectively) was proportional to that previously reported during 1 h whole‐leg heating (+0.62 l min^−1^) (Koch Esteves et al., 2021). These observations together substantiate the impact of volume of heated tissue on the magnitude of hyperaemia evoked by the same type of thermal intervention (whole‐leg > whole‐thigh > quadriceps). Furthermore, in line with the literature, WTH stimulated a two‐fold increase in upper‐leg blood flow without affecting the unheated lower leg or contralateral leg (Koch Esteves et al., 2021). A novel finding was that the 1.4‐fold increase in upper‐leg blood flow during PQH and QH was confined to the directly heated tissues. This interpretation is supported by the observations that hamstrings tissue oxygen saturation – a surrogate for tissue perfusion (Davis et al., 2006) – and hamstring skin and muscle temperature in the experimental leg remained unchanged during QH and that the temperature, skin blood flow and tissue oxygen saturation of the unheated area of the quadriceps (≤18 cm distal to the heated source) was unaffected during PQH (Figure 2). Collectively, these data indicate that tissue blood flow during isolated heating, including a small area of the quadriceps muscles and overlying subcutaneous fat and skin, is regulated in direct response to increases in local tissue temperature.

### 4.2 Interaction among heating modalities and tissue temperature in the regulation of tissue perfusion

A pertinent finding from the present study is that the type of thermal intervention can influence the magnitude of thermal stimulus and hyperaemia. The present study used a hot water‐perfused garment during WTH and QH, and diathermy during PQH. Interestingly, the magnitude of hyperaemia induced by 1 h of PQH was not different from WTH and QH despite only targeting approximately 8% of the upper‐leg's total surface area. Diathermy is known to increase muscle temperature at a depth of ∼3 cm by 3–4°C within 30 min (Benincá et al., 2021; Draper et al., 2013; Garrett et al., 2000; Hafen et al., 2018). In this study, PQH increased muscle temperature from 34°C to 37°C. Although a similar peak quadriceps muscle temperature of ∼37°C was observed in the three protocols, the magnitude of increase was different with WTH, QH and PQH increasing quadriceps muscle temperature by 1.8, 1.5 and 2.7°C, respectively. The thermal stimulus was, therefore, potentially greater during PQH, which could explain the significant hyperaemia despite the smaller heated area. Previous studies which have employed local leg heating and leg cooling following heating or exercise provide strong evidence that tissue perfusion of the temperature‐manipulated region more closely mirrors deep muscle (2−3 cm) temperature in comparison to skin temperature (Caldwell et al., 2016; Chiesa et al., 2016; Gregson et al., 2011; Heinonen et al., 2011; Mawhinney et al., 2013, 2017; Pearson et al., 2011; Sekins et al., 1984). Most striking is that diathermy has been shown to increase thigh muscle temperature from ∼34°C to ∼42°C during simultaneous skin cooling with a concomitant elevation in muscle blood flow from ∼3 ml min^−1^ 100 g^−1^ up to ∼48 ml min^−1^ 100 g^−1^ (Sekins et al., 1980, 1982, 1984). Taken together, these findings indicate that the level of muscle thermal stimulus also impacts the magnitude of rise in tissue perfusion.

Temperature, tissue perfusion and tissue oxygen saturation remained unchanged in the non‐heated limb hamstrings and proximal quadriceps tissues during QH and PQH (Figure 2). Limb tissue perfusion during systemic hyperthermia is postulated to be mediated through local and central thermosensitive mechanisms, working either separately or in tandem (Chiesa et al., 2019; Johnson & Proppe, 1996). The present finding that local tissue perfusion and temperature were unchanged in the non‐heated areas alongside a maintained core temperature aligns with past studies advocating for the predominant role of local thermosensitive mechanisms in hyperthermia‐induced hyperaemia (Chiesa et al., 2016, 2015; Kalsi et al., 2017; Koch Esteves et al., 2021). However, this finding conflicts with past studies that have demonstrated the influence of an augmentation in central haemodynamics, such as a cutaneous vasodilatory reflex drive, during whole‐body and/or local‐limb heating where blood flow increases not only in heated limb but also in the non‐heated or cooled contralateral limb (Caldwell et al., 2016; Heinonen et al., 2011; Johnson et al., 1976; Mallette et al., 2016; Taylor et al., 1984). In those studies, core temperature was regarded as the primary stimulus underlying the global haemodynamic adjustments: a 1°C increase in core temperature is associated with an approximately nine‐fold, 3 l min^−1^, and 35 beats min^−1^ increase in skin blood flow, cardiac output and heart rate, respectively (Chiesa et al., 2019; Johnson & Park, 1979). In support, Heinonen et al. (2011) found that during unilateral calf heating where core temperature remained unchanged, blood flow increased in the heated calf but not in the control, contralateral calf. However, during whole‐body heating where core temperature increased by 1°C, blood flow not only increased in the heated calf but also in the non‐heated, contralateral calf alongside large increases in local vascular conducance (Heinonen et al., 2011). The observed hyperaemia in the unheated contralateral calf supports that core temperature can indeed stimulate a vasodilatory reflex drive (Heinonen et al., 2011). This scenario is different from the present experimental conditions where core body temperature remained unaltered. During the isolated hyperthermia conditions of this study, local thermosensitive mechanisms play a primary role in the regulation of peripheral tissue perfusion. In support of the existence of local temperature‐sensitive regulatory mechanisms, a recent study utilising a 3‐day‐old chick embryo model revealed that after the heart has been arrested with KCl, blood velocity in the vitelline vessel still increased ∼ 3.7‐fold in response to infrared radiation (heat energy) but ceased completely when the heat source was taken away. Hence, the present data together with previous observations in the literature (Manteuffel‐Szoege, 1960, 1969) indicate that a heat‐dependent mechanism can operate in the circulatory system (Li & Pollack, 2023).

The present observations raise the question of why increases in temperature and perfusion were confined to the area directly under the heat source. Heat can be transferred between biological tissues through two pathways: intercellular conductive and vascular convective heat transfer (González‐Alonso, 2012; Khaled & Vafai, 2003). Conduction is the transfer of heat energy through a positive temperature gradient across media in direct contact (Incropera et al., 1996), and is known to be a relatively slow process as it is reliant upon a positive temperature gradient and the thermal conductivity of the surrounding tissues (González‐Alonso, 2012). This pathway is responsible for the transfer of heat from the water‐perfused garment to the upper leg skin during WTH and QH via a positive thermal gradient between the garment and skin. On the other hand, given that the upper leg contains a dense vascular network consisting of arterioles, post‐capillary venules, capillaries and their cellular constituents (Guven et al., 2020), convection – the transfer of heat through the movement of blood – plays a significant role in heat transfer within a limb (Fiala & Havenith, 2015; Incropera et al., 1996). The resultant concomitant increase in internal limb tissue perfusion during local heating further increases convective heat transfer through the various microvessels perfusing the internal tissues, exchanging heat in the capillary beds until it reaches equilibrium with the surrounding tissues (Chato, 1980; Fiala & Havenith, 2015). Whilst the blood perfusing the heated tissues can act as a heat source to the neighbouring unheated tissues (Baish et al., 1986), the large disparity between heated and non‐heated tissue volume as well as the vast network of vessels means that the heat is quickly dissipated in the surrounding unheated tissues and/or heat transfer is very small between the heated and non‐heated areas. This is supported by past studies demonstrating a 5–10°C difference in tissue temperature between the heated tumour and adjacent tissues during targeted tumour hyperthermia, though the distance between the heated tumour and unheated adjacent tissue is unknown (Kim et al., 1977; LeVeen et al., 1976; Suit & Gerweck, 1979). Consequently, the present findings provide evidence that local heating of a limb segment does not cause a heightened tissue perfusion to the proximal non‐heated tissues which quickly dissipate the heat, likely due to a combination of an insufficient thermal stimulus and the human body's ability to maintain thermal homoeostasis when heating is applied to a small segment of the human limb.

### 4.3 Experimental considerations

There are several methodological considerations in this study. First, during QH and PQH, it is not possible to directly measure which tissues are being perfused via the femoral conduit arteries. Consequently, tissue oxygen saturation measures of the heated and non‐heated regions were used to assess regional tissue perfusion. Whilst there is some debate as to whether tissue oxygen saturation data solely reflect muscle perfusion or a combination of cutaneous and muscle perfusion, there is a consensus that it does indeed provide a measure of tissue perfusion (Choo et al., 2017; Davis et al., 2006; Koch Esteves et al., 2021; Pearson et al., 2011). Thus, we are confident in our interpretation of the data. Second, during PQH, baseline skin and muscle temperatures were lower than in the other two protocols as the leg was left exposed in ambient conditions for >1 h whilst protocol 2 was being performed on the right leg. This introduced challenges when making between‐protocol comparisons. Third, during PQH, the electromagnetic waves from the diathermy unit interfered with some of the data recording equipment. Hence, we were only able to collect temperature data by pausing the diathermy unit for 1 min. While this does not directly affect the quality or validity of the present findings, it would have been of great value to have continuously measured the increase in temperature at the onset of heating as diathermy has been shown to rapidly increase muscle temperature (Benincá et al., 2021; Draper et al., 2013; Garrett et al., 2000; Hafen et al., 2018). Fourth, the sum of SFA and PFA blood flow does not perfectly match the measured CFA blood flow. This is likely due to the difficulty faced when attempting to obtain high‐quality images of the PFA, in comparison to other main conduit leg arteries, as the PFA is highly influenced by the individual vessel anatomy (Hussain, 1997; Tomaszewski et al., 2017; Koch Esteves & Chiesa, 2021). Nonetheless, this potential limitation does not affect the interpretation and conclusion of the present findings. Lastly, whilst female participants were requested not to attend during menses, the cycle phase and use of hormonal contraceptives were not controlled for. Thus, whilst we acknowledge that not obtaining cycle and contraceptive information is a limitation, we believe that our data and findings remain valid and reliable.

### 4.4 Summary

Passive WTH, QH and PQH evoked 1.4−2‐fold increases in leg blood flow which mirrored the increases in local tissue temperature. Moreover, the QH and PQH protocols increased tissue perfusion to their respective heated regions without affecting perfusion and tissue oxygen saturation of the non‐heated area within the same segment, likely due to an unchanged tissue temperature. These results therefore indicate a strong relationship between local tissue temperature and perfusion even in a small area of a human lower limb. Ultimately, this close relationship, regardless of the volume of the heated limb, further highlights the significance of local thermosensitive mechanisms in the regulation of tissue perfusion during hyperthermia.

## AUTHOR CONTRIBUTIONS

This study was performed at Brunel University London, Uxbridge, UK. Nuno Koch Esteves, Jeneil McDonald and José González‐Alonso conceived and designed the research. Nuno Koch Esteves, Jeneil McDonald and José González‐Alonso acquired the data. Nuno Koch Esteves analysed the data. Nuno Koch Esteves and José González‐Alonso interpreted the data. All authors have read and approved the final version of this manuscript and agree to be accountable for all aspects of the work in ensuring that questions related to the accuracy or integrity of any part of the work are appropriately investigated and resolved. All persons designated as authors qualify for authorship, and all those who qualify for authorship are listed.

## CONFLICT OF INTEREST

The authors declare no conflicts of interest.

## FUNDING INFORMATION

No funding was received for this work.

## Data Availability

The raw, unidentified data collected throughout this study will be made available via Brunel Figshare, an online data repository database.
